# The ‘lived experience’ of palliative care patients in one acute hospital setting – a qualitative study

**DOI:** 10.1186/s12904-018-0345-x

**Published:** 2018-07-06

**Authors:** Anne Black, Tamsin McGlinchey, Maureen Gambles, John Ellershaw, Catriona Rachel Mayland

**Affiliations:** 1 Palliative Care Institute Liverpool, Cancer Research Centre, University of Liverpool, 200 London Road, Liverpool, L3 9TA UK; 2 Royal Liverpool and Broadgreen University Hospitals NHS Trust, Prescot Street, Liverpool, L7 8XP UK

**Keywords:** Patient experience, Narrative research, Palliative care, Hospital, Qualitative

## Abstract

**Background:**

There is limited understanding of the ‘lived experience’ of palliative care patient within the acute care setting. Failing to engage with and understand the views of patients and those close to them, has fundamental consequences for future health delivery. Understanding ‘patient experience’ can enable care providers to ensure services are responsive and adaptive to individual patient need.

**Methods:**

The aim of this study was to explore the ‘lived experience’ of a group of patients with palliative care needs who had recently been in-patients in one acute hospital trust in the north-west of England.

Qualitative research using narrative interviews was undertaken, and data was analysed using thematic analysis. A sample of 20 consecutive patients complying with the inclusion/exclusion criteria were recruited and interviewed.

**Results:**

Patient Sample:

Of the 20 patients recruited, there was a fairly equal gender split; all had a cancer diagnosis and the majority were white British, with an age range of 43–87 years.

Findings from Interviews:

Overall inpatient experience was viewed positively. Individual narratives illustrated compassionate and responsive care, with the patient at the centre. Acts of compassion appeared to be expressed through the ‘little things’ staff could do for patients, i.e., time to talk, time to care, humanity and comfort measures. AHSPCT involvement resulted in perceived improvements in pain control and holistic wellbeing. However, challenges were evident, particularly regarding over-stretched staff and resources, and modes of communication, which seemed to impact on patient experience.

**Conclusions:**

Listening to patients’ experiences of care across the organisation provided a unique opportunity to impact upon delivery of care. Further research should focus on exploring issues such as: why some patients within the same organisation have a positive experience of care, while others may not; how do staff attitudes and behaviours impact on the experience of care; transitions of care from hospital to home, and the role of social networks.

## Background

‘Person centred’ approaches to care delivery have been promoted as a core part of service design within the National Health Service (NHS) [1]. Crucially, person centred care promotes a care environment that is respectful, compassionate and responsive to the needs of individuals [2]. This is not a novel idea as the person centred ethos can be seen echoed in the core principles and values of the NHS; “[the NHS] touches our lives at times of most basic human need, when care and compassion are what matter most” [3]. Whilst this may be an attractive concept to underpin health care delivery policy, the term has been criticised for being applied without clarity of definition, causing subsequent discourse around the subject to be ‘woolly’, particularly with regard to informing actual care delivery [4].

A recent high profile review of care delivery in hospitals has shown that a lack of openness and compassion led, at times, to care that was “totally unacceptable and a fundamental breach of the values of the NHS” [5]. Furthermore, the Neuberger review highlighted a lack of ‘patient centred’ care and openness around decision making as barriers to good care [6]. A failure to engage meaningfully with patients may result in an approach to care delivery that ‘does to’ rather than ‘works with’ patients; privileging the perspective of healthcare professionals and clinically focused outcomes [7]. Indeed, a lack of compassion from health care providers has been cited as a major reason for dissatisfaction with the care that patients receive [8].

Failing to engage with and understand the views of patients and those close to them, has fundamental consequences for future health care delivery. Both government policy/guidance and the research literature continues to emphasise the importance of exploring the ‘patient experience’ in order to support service providers to provide care that is responsive and adaptive to individual patient need – ie person centred [2, 9–12]. By actively seeking the views of patients and families, the potential to ensure that these views are placed at the centre of service provision is enhanced. This perspective sits in accordance with the overarching values of the NHS Constitution [3] as well as National Guidance for End of Life Care [10, 12, 13]; therefore engaging service users should form part of ongoing service improvement strategies.

Predominantly however, assessing the ‘user experience’ has centred on measuring ‘satisfaction’, with a focus on comparison and monitoring. Some commentators suggest that current widely used approaches for measuring ‘satisfaction’ may not be sufficiently grounded in the values or experiences of patients, thus raising serious questions about the validity of the concept as a way of eliciting what is important to patients and the care they receive [14, 15]. In recent years assessment of the performance of healthcare organisations has begun to move beyond examining clinical care alone, to considering and embracing ‘patient experience’ as an important indicator of quality [9].

So how can we best uncover the views of patients who receive care in our NHS organisations, to better understand how well it meets their needs? Patient experience is complex and multifaceted, and requires more in depth methods to explore how patients and families experience the care they receive [9]. Taking time to actively engage patients to find out what is really important to them has the potential to unlock a richness of information not possible solely through ‘satisfaction’ questionnaires alone [16].

Much of the recent focus of both the media and the academic literature has been on the perceived deficits in care delivery for hospital in-patients nearing the end of life and their relatives and carers [6, 17]. We therefore chose to focus this study on a group of hospital in-patients who had life limiting illness and who were potentially nearing the end of life. In order to identify a suitable group of patients, we focused on inpatients who had received input during their stay from members of the Academic Hospital Specialist Palliative Care Team (AHSPCT) in one acute hospital trust in the North-West of England. The AHSPCT is an advisory service which takes referrals from across the hospital for patients with identified specialist palliative care needs. The role of the service is to assess patients’ holistic needs in order to optimise comfort, well-being and quality of life, in the presence of incurable, advancing illness. The AHSPCT is a multi-professional team, and includes doctors, specialist nurses and allied health professionals.

## Methods

The aim of this study was to explore the ‘lived experience’ of a group of patients with palliative care needs who had recently been in-patients in one acute hospital trust in the north-west of England.

Exploring the lived experience required a phenomenological approach whereby participants were encouraged to recount their experience, allowing issues that held most personal importance to them unfold. This approach allows the researcher ‘enter the patients world’, promoting understanding of their experience from the patients’ perspective [18]. In-depth narrative interviews were undertaken using a conversational approach where patients were encouraged to direct and shape the discussion in accordance with their own experiences, views and particular concerns [19, 20], rather than responding to a pre-determined agenda.

### Procedure

#### Identification and recruitment of patients

In order to promote the potential to sample a range of experience, a consecutive sample of 20 patients who had been referred to the AHSPCT were recruited to take part. Recruitment was coordinated by the main researcher (AB). AB, female, is a Clinical Nurse Specialist with the AHSPCT, who was seconded for 1 year to undertake this research project.

During the recruitment phase, AB attended the morning ‘run through’ meeting within the AHSPCT attended by the multi-disciplinary team, to prompt identification of patients who may be ‘eligible’ for this study. Patients were considered ‘eligible’ if they met the following inclusion criteria:

- Hospital inpatient > = 18 years of age
- Referred to the AHSPCT and seen on at least two occasions;
- Due to be discharged from hospital.

Patients were not approached for this study if the following exclusion criteria applied:

- Hospital inpatient < 18 years of age;
- Recognised to be in the last few days or hours of life;
- Unable to provide fully informed consent to participate;
- Died prior to discharge;
- Unable to communicate in English.

### Information and consent

Potential participants were initially approached by a member of the clinical team, who informed them that this study was being conducted. If the patient expressed interest, they then met with the researcher (AB), who gave them a Patient Information Sheet (PIS) along with verbal information and offered the opportunity for questions. If the patient was agreeable, a mutually agreed date/time and place was arranged to conduct the interview following discharge from hospital. AB then checked their agreement to participate prior to undertaking the interview, and a consent form was signed by the participant.

### Interviews

The interviews were conducted by the researcher (AB) in the patients’ home following discharge. The researcher began the interviews with an open question:

‘Thinking back to x number of days ago when you came into hospital, can you tell me everything that has happened’.

A topic guide of ‘prompts’ was also created to support this process. For example, prompts such as ‘tell me more about’, ‘can you remember specific examples?’ and ‘how did you feel about that?’ were used in order to elicit more detailed responses where this did not occur more naturally from the conversation. The interviews were conducted between October 2015 and September 2016.

It was important to consider issues of potential bias within the research process, for example the balance of power in the relationship between patients and the researcher [21, 22]. Considering this, the interviews were conducted in a place where the patient felt comfortable, and the researcher kept a field note diary to document thoughts and feelings in order to aid ongoing reflection. In addition a distress protocol was available should the patient become distressed during the interview.

### Analysis

Each interview was transcribed verbatim, and transcripts were analysed using Thematic Analysis, facilitating exploration of how people ascribe meaning to their experiences in their interactions with the environment [23]. The analysis process began at the interview stage, with the researcher keeping a field note diary of thoughts, feelings and emotional responses to the interview process and content. The process of analysis was cyclical and iterative in nature. Transcription further promoted familiarisation with the data and generation of initial emerging themes. The transcripts were also analysed in conjunction with the original recordings, so that the researcher became fully immersed in the data [23]. Against each transcript, the main researcher (AB) made initial notes documenting any observations, questions and interpretations that arose from the reading and re-reading of the data. AB then coded each transcript and made an initial narrative summary of the key themes for in-depth discussion with the wider team (TM and CM). TM and CM also independently analysed 5 transcripts (20%) to gain first-hand experience of the words of participants, giving the potential for a richer interpretation. Where appropriate, consideration of relevant published literature further enhanced the evolving interpretation.

## Results

### Final sample

**Fig. 1:**
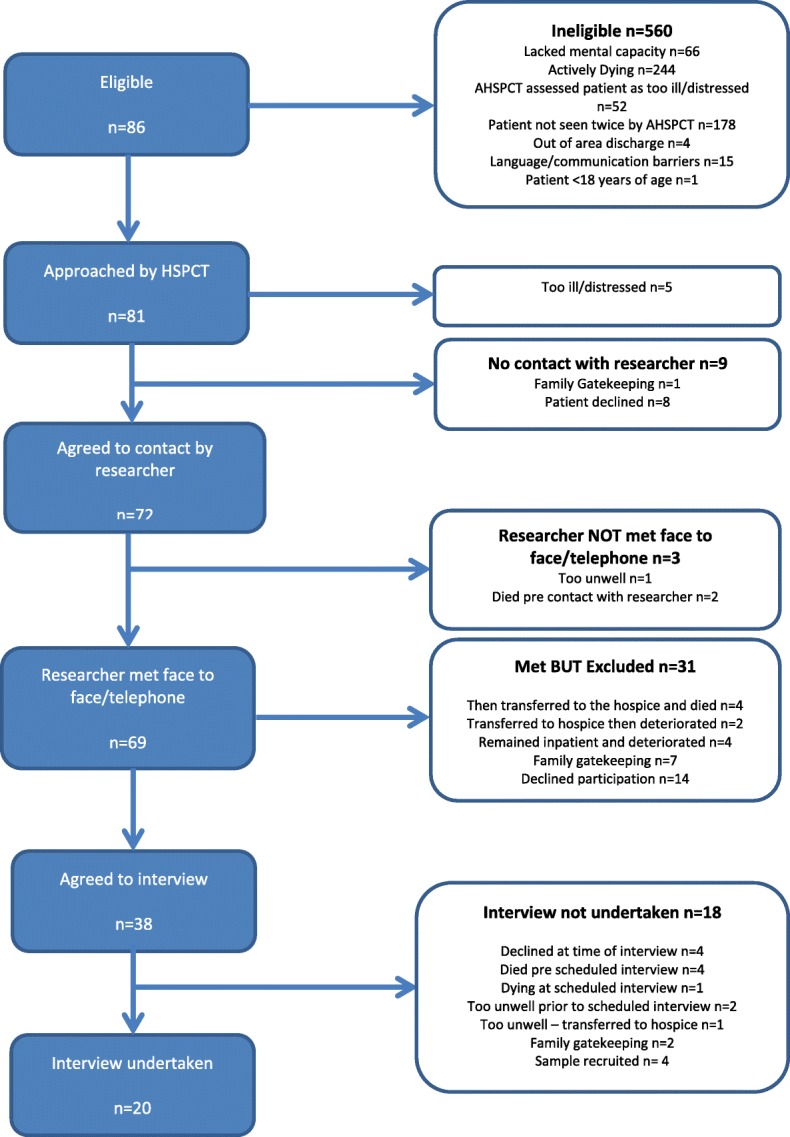
Flow Diagram for Recruitment

A total of 20 interviews were undertaken (see Fig. 1 for recruitment flow diagram) lasting between 15 min and 90 min, with a median time of 41 min.

As a result of the complex and palliative nature of the patient cohort, over half (53% *n* = 296/560) initially referred to the AHSPCT were either ‘too ill’ or ‘dying’ at the point of referral, meaning they were not eligible for inclusion. However, many patients who were approached for inclusion expressed interest in taking part in the study; of the 81 patients initially approached only 26 (32%) expressly declined. Thirty five patients (43%) initially showed interest but were unable to be recruited for the following reasons: deteriorating condition (*n* = 11); subsequent death (*n* = 10); family ‘gate keeping’ (n = 10); and the required sample had been reached (*n* = 4). The interviews took place no longer than 10 days following discharge home; 14/20 interviews took place within 6 days of discharge. Table 1 provides a summary of the demographic details of participating patients.

**Table 1:** Demographic Details

Total No: Participants	20
Male	11 (55%)
Female	9 (45%)
Age Range	43–87 years
Diagnosis	20 cancer (100%)
Ethnicity	19 White British (95%) 1 Any other ethnic group (5%)
Median days - recruitment to Interview	6 days (IQR 5–7 days)
Median days - Interview to Date of Death (*n* = 17^a^)	63 (IQR 35–218 days)

^a^3 patients still alive at close of data collection period

### Findings from interviews

Four overarching themes were generated from the interview data and these are presented below.

### Making Time – Taking Time

It was clear from the narratives that participants in this study were acutely aware of the pressures on the staff that were looking after them, including the busyness of the wards, and staff shortages:

*“…sometimes they were run off their feet. They can’t always come so you don’t get bad tempered or anything, you just have to wait and know that they will come.” (Betty).*

*“they’re very, very busy and they’re trying to fit you in and decide what’s the best thing to do for you and they haven’t got time to do, I wouldn’t even call it value added, but to just communicate to you to say, ‘right Mr P, this is what we plan to do and this is why we’re doing it. There was none of that...because they are so busy and they haven’t got time and resource in place to provide that information to you” (Bill).*

Against this backdrop, the views of the participants highlighted how the mode and manner of communication and information giving, including the number of HCPs involved and the level of engagement, could further negatively impact their experience:

*“...I saw four different teams, you know what I mean, so you do lose track that is; who and names (sic)...that was one of the problems I had anyway.” (Gerry).*

*“That [lack of information] leaves you feeling as though...do they know any more, that they don’t want to tell me? ...or is [it] a matter that they just don’t know what’s going on?” (Bill).*

For some, it was perceived that it was not just busyness that meant that staff were less attentive than they would have liked, but individual differences in the way different staff approached their roles:

“*Well it was sort of nurses, I mean, erm there was some of them were, it’s hard to say, some of them were a lot better than others .. but there was others not so good; they would sit round chatting and things like that when there was, you know, basically, work to be done .. I mean you waited every night till nine o’clock to see which nurse .. was gonna come on and .. you know if they were good nurses .. you would have no problems” (Harry).*

Understandably then, staff that went the extra mile to make time in their busy schedules and to take time to treat these patients as individuals, were highly valued:

*“…it’s just little things…that make a difference...they wanted to be there, they wanted to care. You could tell that they wanted to care…and they made time for me…they just seemed to care…to want to be there and help...they wanted to listen to what I have to say and understand how I feel …one particular nurse, she just said to me one night, you’re not you’re normal self…do you need a hug? And I said, “Yeah, I do actually”. So she gave me a hug and you know, she hugged me for a while until I was ready to stop having a hug...*” *(Tilly).*

*“nurses used to sit with me, not only about the medication, but they used to sit with me and listen to problems, about my health and what was going on and they used to sit with me for quite a while” (P7).*

### Experiencing and relieving pain

For some patients their in-patient stay was characterised by their experience of pain, and it was often what they remembered most about being in hospital.

*“Erm, it’s like you know if someone, they had like, erm, wood and paper and everything and they put a match to it and it went aflame, that’s the way I feel, ya know when it hits my right leg…that’s how the pain was, and I felt like a fire had gone off inside me.” (Betty).*

Where physical pain was not dealt with in an appropriate and timely manner, this was highlighted as having the potential to negatively impact the patient experience:

*“…they [nurses] gave me paracetamol thinking it would help and I just sat up in the chair, I’d say for about three nights... they couldn’t give me anything stronger because I wasn’t written up for it so I was sat in the chair...trying to stop the pain and just ended up sitting up all night watching TV… just watching the clock until nine o’clock, until they came round with the medication” (Sadie).*

*“Sometimes we ask for medication and they’ll say I’ll get it for you, and you’d end up getting it eventually when they’d come round with the trolley two hours later...” (Bob).*

When this was attended to however, the therapeutic value of this for patients made all the difference. The act of attending to patients’ pain relief appeared to embody compassion, care, dignity, and being valued as a human being:

*“That was great, and somebody’s on your side, I can remember her coming up to me, whispers “I got you some more” [medication], oh thank God, yeah…*” *(Ritchie).*

Interestingly, although initial anxiety was reported by some around whether the involvement of the Academic Hospital Specialist Palliative Care Team (AHSPCT) meant imminent death, it was their involvement, particularly with regards to pain management, that was highlighted as having had a positive impact:

*“Oh the pain relief, they [AHSPCT] were absolutely marvellous…it was like someone waving a magic wand because after I’d seen them for a few occasions, about three times, er, I just, the next time they came to see me, I said it was the first time that I’d slept properly in about six weeks.*” *(Sadie).*

### Loss of control and loss of self

Central to many patient stories, was the sense of ‘struggle’; seeking to find sense and meaning in their lives in the face of an uncertain and changing future with a life limiting illness:

*“I didn’t know I was dying seven weeks ago...eight weeks ago I just had a bad back. I was actually working and doing stuff and planning my life and wanting to get better, expecting to get better, but now I’m dying and I’m not expecting to live, so I don’t...I wanna understand what’s happening to me and I wanna understand what’s the likely scenario but there’s a part of me that’s terrified. I’m terrified of like being in agonising pain. I’m terrified of like losing meself (sic) to the pain; the pain steals your personality.” (Tim).*

Patients also described feeling ‘labelled’ by their illness, which in turn poses a challenge to their sense of ‘self’ and ‘identity’:

*“Terminal, you know what I mean. Er, you do seem to feel a bit, a little bit different.*” *(Terry).*

Linked to this, some patients described the ‘contagiousness’ of cancer, and almost a sense of isolation, from having the ‘label’ of a cancer diagnosis:

*“I suppose in the back of your mind...cancer is contagious...don’t you, sounds silly doesn’t it? ...I suppose that’s were you, er you think it’s, it’s a horrible word cancer, but it means a lot of things doesn’t it?” (Charlie).*

For some the hospital environment provided a ‘secure’ and ‘supportive’ environment during this time of flux, however once discharged home, patients described feeling ‘alone’ and less supported:

*“...when you come home you’re very much left to your own devices...now I’m in need of a bit of help and support...I feel as though I’m being provided with a poor...well not a poor service, but a limited service” (Bill).*

### Burden versus benefit of treatment interventions

From these patient stories, a picture emerged of wrestling with choices and decisions regarding treatment options. This illustrates the subjective values placed on ‘life’; quality of life or the battle to survive at any cost.

*“I know I’m not gonna get better, and I thought, why do it, you know? Why put me through anything that’s intrusive at all? I really don’t see the point; I really don’t.” (Wendy).*

*“…when you have a days like the last couple of days I’ve just felt ill…it’s difficult to wanna like, battle on…fighting the sickness is horrible…I’m not sure if I wanna go back, to go back to radiotherapy though. I’m not sure I’d like it or trust it. I don’t know how making me feel this ill; can be doing me any favours.” (Tim).*

The following patient quote illustrates the tensions that can arise when HCP and patients’ perceptions of the focus of care are not aligned, impacting on patient choice, autonomy and dignity and shared decision making:

*“…it changes when you become terminal. I could understand [considering all treatment interventions] before because then there is a real good case for it…once you go into the terminal thing then it’s a case of not so much…it’s a case of what can…make it better for now? And if the blood thinners was making me a lot worse so to me, my personal opinion, in that situation was let’s just stop them. It might not have been somebody else’s [wish] but nobody was actually saying…they were saying “This is what’s going on” but [not asking] “what do you want to do?”” (Terry).*

The following patient account highlights that when HCP ‘take on board’ what the patient wants, and work in partnership, this can alleviate the ‘tension’ and provide therapeutic benefits. This in turn impacts on patient autonomy, dignity and comfort, reinforcing the importance of active listening and shared decision making:

*“[I felt] Jubilant…because like I say over a year and somebody’s listened, and they’ve gone away, they’ve sorted it all out, done what they promised they’d do you know like oh we’ll get it sorted, and we’ve heard that so many times, and no they did exactly what they said they’d do…that’s all I could ask that somebody would listen, and take on board what the patient wants, as well as what the doctor’s experiences are, obviously a two-way street, but when it comes to pain the patient knows what pain they’re in, not the doctor.” (Ritchie).*

## Discussion

This study has generated important information on the way in which patients’ experience care currently, providing an opportunity for the acute hospital to generate recommendations, to consider how results from this study may inform future service design, education, training and resource utilisations. The results of this study illustrate that overall the in-patient experience was viewed positively for most patients, with accounts illustrating compassionate and responsive care. Challenges were highlighted, however, with regard to over stretched staff and resources, along with individual differences in the attitudes of staff, which was reported to have negatively impacted the experience of care for some patients. Whilst this study was undertaken in one acute hospital, these findings are likely to be of interest to all providers of in-patient care, as many of the themes and issues highlighted here may also resonate with those care services.

Where care delivery was timely, responsive, well led and compassionate, however, this appeared to contribute to patients feeling safe and valued as individuals rather than being ‘processed’ as commodities; a view reinforced in the literature and recent policy documents [10, 24, 25]. In this study, acts of compassion were experienced through the ‘little things’ that staff could do for patients such as; making and taking the time to talk, to care and to display characteristics of humanity. Indeed, one of the main components of ‘good care’ has been highlighted as feeling that ‘you matter’ [26]. This perspective supports the view that the smallest details of the patient experience can be the most meaningful [27]. The NHS is under relentless pressure to improve efficiency and throughput; however it is an imperative that the patient remains at the forefront of any improvement strategy [2].

For patients’ in this study, modes of communication could have both positive and negative impacts on the patient experience. In particular, what information was given and how it was delivered appeared to impact on patients’ understanding of services involved, their condition and the overall plan of care. Evidence suggests “effective communication is the core of every helping relationship, and listening is the foundation of every medical and social service interaction” [28], p57. Accounts from this study reinforce that when HCP’s were able to ‘connect’ with patients beyond the ‘physical’ contact, this fostered a powerful sense of genuine human presence and care; effective communication, engagement and active listening, should be reflected within the culture of care in the organisation [29]. In recognition that ‘dignity enhancing’ or ‘dignity preserving’ care for palliative care patients is vitally important, the use of interventions such as the ‘dignity model’ has been highlighted as one way to ensure a person-centred approach in the acute hospital setting; promoting patient autonomy and recognition of the person as an individual [30].

For many patients in this study, pain appeared to be a major concern throughout their in-patient episode; a finding supported by previous studies [31–33]. Stories from this study reinforce the ‘threat’, highlighted by Pringle et al. [30], that untimely and unresponsive symptom assessment and control can be to patient dignity. For example patients described the seemingly all-encompassing nature of pain and the very real distress this caused when it was unremitting and unresolved. Specifically, some patients described ‘a significant period of waiting for assessment and administration’ of pain medication, impacting on their sense of dignity and wellbeing. Poignantly, patients described their relief when they felt that their pain was finally being attended to, underlining the significance of pain control to a patient’s sense of being cared for and valued as a human being. The role of the AHSPCT was specifically highlighted in this regard, where despite initial uncertainty and anxiety from some patients associated with their understanding of the role of the AHSPCT [31, 34, 35] as noted in previous studies [30, 31, 36, 37], their involvement resulted in improvements in pain control and holistic wellbeing.

Throughout this study, patients’ described the ‘struggle’ of living with a terminal illness, and the effect this had on their sense of self and life as they knew it before their diagnosis. This was a very important issue for patients, as their sense of ‘self’ had been ultimately changed, forcing them to renegotiate this in the face of uncertainty: “Death forces us to give an ultimate meaning to life and thereby transcend the apparent absurdity and meaninglessness of life in the face of death” [38].

Patients described feeling ‘different’ following their diagnosis, which echoes previous studies where the ‘stigma’ of cancer can have a negative impact on a patients sense of self, resulting in a ‘renegotiation’ of identity within the new context of their diagnosis [39]. It has also been suggested that over time the ‘label’ of a terminal illness can preclude ‘sustaining self-images’ resulting in ‘diminished self-concept’, as well as a fear of becoming a ‘burden’ to relatives as they readjust to the ‘real world’ [40]. This echoes with findings from this study, where for example despite the ‘hustle and bustle’ the hospital provided a ‘safe haven’ during this uncertain time [41], where patients could navigate and readjust within their ‘renegotiation’ of identity, self-worth, dignity and self-respect.

For some patients in this particular study, the distress prompted by this time of uncertainty extended beyond their inpatient admission. Some patients reported feeling ‘alone’ following discharge, indicating the potential for ongoing distress and need for additional support at this time. This resonates with the idea that ‘structures’ that underpin everyday life (such as social networks and relationships) can be ‘disrupted’ in light of serious chronic illness [42]. The ‘chaos narrative’ [43, 44] offers us another perspective that resonates with this study, for example the challenge of loss and adjustment faced by study participants when leaving the safe confines of hospital to return to the’ real world’. Reinforcing the importance that care services should not ‘end’ at the point of discharge, ensuring that patients can be sufficiently supported.

Johnson suggests ‘living with dignity’ is bound up in the individual’s sense of identity; through having one’s human value acknowledged, irrespective of circumstances, ‘personhood’ and ‘self-worth’ [45]. Johnson also highlights the risk to dignity at the end of life (EOL) as health deteriorates being particularly concerning [45]. Therefore, as health professionals, it is crucial that we consider how we respect these views in our conduct with others, ensuring that our interactions are dignity enriching [45], seeing the ‘person’ in the patient, rather than merely their illness. This perspective is also highlighted by Chochinov [46] and Johnson [47], who describe the Patient Dignity Question (PDQ) as a means by which HPCs may enhance person-centred care, for people with palliative care needs in an acute hospital.

### Strengths and limitations

This study provided a unique opportunity for one NHS organisation to explore what matters to patients with a life limiting illness, in the context on their in-patient stay. The approach that was taken, through listening to ‘patient stories’, reflects the traditions of hospice and palliative care, by giving time and space to listen and gain a greater understanding from the patients perspective [48].

However it has been recognised that involving patients with a palliative illness in research studies poses its own ethical and moral challenges. In this study for example due to the vulnerability of the patient population, some were unable to be involved as they deteriorated or died prior to or after discharge from hospital. Despite ethical and methodological debates regarding the ‘morality’ and ‘appropriateness’ of involving this cohort of patients in this type of research [49], it was evident throughout recruitment, that patients had a desire to take part. Indeed there is growing evidence to suggest that in fact, palliative care patients do have a desire to take part in research [50, 51]. This adds to growing literature, critiquing the potentially constraining ethical guidelines, prompting the question of whether it is ethical to prohibit patients the chance to contribute to research [52, 53].

Also of note was that the majority of interviews took place within the last two months of the patient’s life (17/20 had died by the end of the data collection period: October 2015 – September 2016). This is interesting given the reticence to involve patients in research as they are approaching the end of life, due to the assumption that it is an unwelcome burden for them at this time [46]. The inclusion criteria of this study however excluded patients that remained in hospital. It could be argued that this approach limited participation, possibly denying the opportunity for other palliative care patients to share their experiences and potentially silencing their voices. In addition, the sample was homogenous in terms of ethnicity and all had cancer, therefore future studies may seek to explore the views of a wider patient population, including patients that do not have a life-limiting illness. Interestingly, the referral criteria for the AHSPT are not limited to patients with a cancer diagnosis, yet these patients made up the total sample population for this study.

The issue of ‘gatekeeping’ was also important to consider, as for ten patients in this study family members specifically requested that the patient not be approached. Reasons for this included perceptions that the patient was too unwell, too tired, or it was ‘not the right time’ to be approached, despite some patients agreeing to meet or have contact with the researcher. However, there were examples where family ‘gatekeepers’ became part of the process [54], by facilitating access to the patient and by their presence in the interview itself, potentially shaping the stories that were being told. It is important to be mindful of these influences when undertaking this kind of research.

## Conclusions

Despite the acknowledged organisational pressures, these patient narratives highlight the importance of concepts such as kindness, compassion and dignity; taking the time to ‘care for patients’ rather than time to ‘do to patients’, taking the time to listen to what is most important and taking the time to respond to the patient as an individual. When the patients’ voice is heard and healthcare professionals ‘see the person behind the name’ rather than the illness, this provides opportunities for relationships to be built based on trust, confidence and mutual respect. This ultimately impacts on the patients’ experience of care, and their perception of self-worth and identity and sense of dignity [46, 47]. The palliative nature of illness reinforced the ‘preciousness’ of time, underlining there is ‘one chance to get it right’ [55]. Having listened to our patients it is time to learn and change; this study has provided an opportunity for the ‘patient voice’ to be heard and the individual patient experience to be explored. Further research should focus on exploring issues such as: why some patients within the same organisation have a positive experience of care, while others may not; how do staff attitudes and behaviours impact on the experience of care; transitions of care from hospital to home; the role of social networks.

## Abbreviations

AHSPCT: Academic Hospital Specialist Palliative Care Team
EOL: End of Life
NHS: National Health Service
PIS: Patient Information Sheet
